# Nutraceuticals in osteoporosis prevention

**DOI:** 10.3389/fnut.2024.1445955

**Published:** 2024-10-02

**Authors:** Livia Roseti, Giorgia Borciani, Francesco Grassi, Giovanna Desando, Laura Gambari, Brunella Grigolo

**Affiliations:** RAMSES Laboratory, Rizzoli RIT-Research, Innovation & Technology Department, Istituto di Ricerca Codivilla Putti, IRCCS Istituto Ortopedico Rizzoli, Bologna, Italy

**Keywords:** osteopenia, osteoporosis, nutraceuticals, prevention, lifestyle, bone health

## Abstract

Nutraceuticals are gaining popularity as they can contribute to bone health by delaying the onset or slowing down the progression of pathological bone loss. Osteoporosis’s bone loss is a concern for older adults and a crucial aspect of aging. Maintaining healthy bones is the key to living a full and active life. Our review explores the current knowledge on the role of nutraceuticals in preventing osteoporosis by focusing on three main aspects. First, we provide an overview of osteoporosis. Second, we discuss the latest findings on natural nutraceuticals and their efficacy in reducing bone loss, emphasizing clinical trials. Third, we conduct a structured analysis to evaluate nutraceuticals’ pros and cons and identify translational gaps. In conclusion, we must address several challenges to consolidate our knowledge, better support clinicians in their prescriptions, and provide people with more reliable nutritional recommendations to help them lead healthier lives.

## GRAPHICAL ABSTRACT

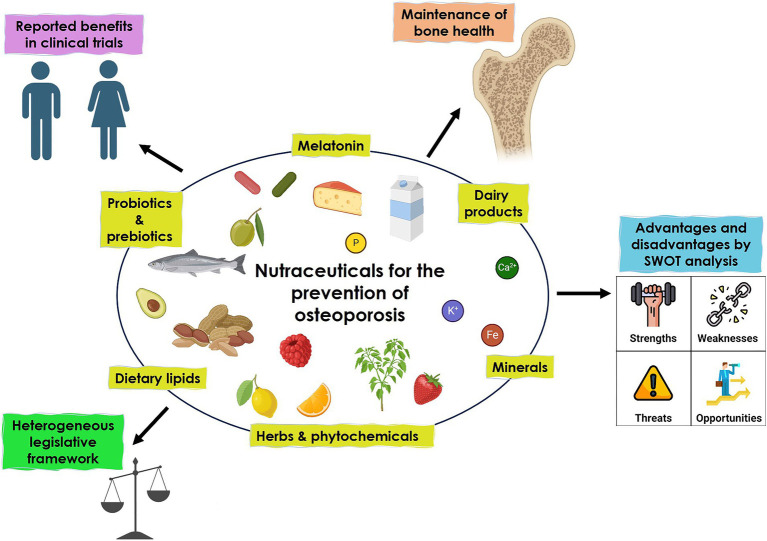

## 1 Introduction

Osteoporosis is a common, debilitating, chronic, progressive, systemic, and metabolic skeletal disease. Bone loss is not just a concern for older adults but a crucial aspect of aging. Maintaining healthy bones is the key to living a full and active life (1–3).

Osteoporotic patient management carries significant health, social, and economic burdens on countries with an increase in the population’s average age. Financial costs are direct, especially concerning treating osteoporotic fractures, and indirect, such as functional disability and loss of productivity. Bone loss prevention can significantly diminish the health care costs and improve the quality of life (4). Available therapeutic options are effective but may present side effects, which can cause discontinuing therapy (5). Moreover, a part of the high-risk fracture population does not get proper care due to cultural and socio-economic factors and a recent diffused skepticism towards official medicine (6).

Several compounds that fall under the definition of nutraceuticals can complement pharmacological treatments aimed at increasing bone mineral density and preventing bone tissue loss (7, 8). Our literature review will highlight the latest developments in natural nutraceuticals that promote bone health and prevent bone loss. Lastly, we will analyze nutraceutical pros and cons, with a focus on their pros and cons.

## 2 Osteopenia and osteoporosis

### 2.1 Bone

Bone is a mineralized connective tissue exerting essential functions: muscle attachment and locomotion, soft tissue support, vital organ protection, bone marrow harboring, calcium (Ca) and phosphate (H_3_PO_4_) storage, and homeostasis (9). Mature bone tissue includes an abundant calcified extracellular matrix (ECM) that supports and interacts with the resident cell types contributing to bone development and maintenance: osteoblasts, osteocytes, osteoclasts, and bone lining cells.

Mesenchymal stem cell (MSC)-derived osteoblasts serve for bone formation; osteocytes are terminally differentiated mature osteoblasts embedded in the mineralized bone, no longer involved in bone formation, but helping control the remodeling process. Osteoclasts, derived from hematopoietic precursors, degrade mineralized ECM (bone resorption). Bone lining cells are quiescent osteoblasts localized at bone surfaces, where bone resorption and bone formation do not take place (9).

Besides cells, the ECM comprises a nanostructured organic phase rich in collagen (mainly type I collagen, mucopolysaccharides, and water), which provides flexibility and tensile strength, and an inorganic mineral phase presenting nanocrystalline hydroxyapatite (Ca and H_3_PO_4_), which ensures bone mechanical rigidity and compressive strength (10–12).

### 2.2 Bone remodeling

Resorption and formation are the two main phases of bone remodeling involving mechanical and chemical signals together with systemic and local endogenous factors that can act synergically or antagonistically. Dis-regulations within this complicated functional network can handle pathological conditions or diseases and thus be the target of the treatment (13, 14).

The bone remodeling cycle comprises five steps: activation, resorption, reversal, formation, and termination/quiescence. Osteocytes detect bone matrix damage and communicate with the other cells through their network of dendrites to start bone remodeling process (16, 17). During activation, osteoclast precursors differentiate towards mature osteoclasts, which attach to the ECM. This way, osteoclasts expose the bone surface and isolate the resorbing compartment from the surrounding bone (15). During resorption, osteoclasts degrade the bone ECM and, in the end, undergo programmed cell death (18). Osteoclasts may either secrete cytokines or act via a regulatory surface receptor to release osteogenic signals to prompt bone formation (19, 20). The reversal phase switches to osteoblast-mediated bone formation, promoting new bone deposition. In the formation phase, pre-osteoblasts continue their commitment toward differentiated cells, secreting molecules to aid bone formation. In the termination/quiescence phase, remodeling process concludes with an equal amount of resorbed and newly formed bone (21, 22). After mineralization, osteoblasts may undergo apoptosis, become bone-lining cells, or differentiate into osteocytes. The bone surface undergoes a new cycle.

### 2.3 Risk factors

Risk factors can be unmodifiable or modifiable or associated with concomitant diseases or the use of specific drugs that can cause bone loss (23).

The main non-modifiable risk factors are: (i) age, osteoporosis is a typical disease of aging; (ii) gender, women are more susceptible to osteoporosis than men due to a lighter and thinner skeleton (23); (iii) menopause, the sharp decline in estrogen levels after menopause and longer average lifespan contribute to increased risk; (iv) genetics, numerous loci are associated with bone mineral density (BMD), and others are related to bone shape, geometry, and microarchitecture (24); (v) ethnicity, osteoporosis prevails in Caucasian and Asian populations than in African and Hispanic ones.

The main modifiable risk factors concern healthy lifestyle habits and often have essential consequences on the gut microbiota (GM) (25). Other modifiable risk factors are: (i) insufficient Ca consumption and hypovitaminosis D that can lead to secondary hyperparathyroidism, which increases bone resorption (26); (ii) low intakes of fruits and vegetables can deprive the body of essential nutrients like magnesium (Mg), potassium (K), and vitamin K; (iii) consuming excessive proteins, sodium, and caffeine can cause Ca loss through urine; (iv) excessive alcohol consumption can reduce new bone formation and increase the risk of fractures; (v) tobacco smoke affects the receptor activator of nuclear factor kappa-light-chain-enhancer of activated B cells (NF-kB) and RANK-RANKL-OPG pathway, directly and indirectly influencing BMD and intestinal microbiota (27); (vi) being excessively thin and having a slight build can increase the likelihood of osteoporosis. Osteoblasts produce receptor activator of nuclear factor kappa beta RANKL, and its binding to RANK supports osteoclastogenesis. Osteoblasts also secrete OPG, which competes with RANK for RANKL, inhibiting osteoclastogenesis. A sedentary life facilitates bone loss, the opposite of low-to-moderate physical activity (28); the skeleton reacts to the reduction of the forces applied by the muscles on the bone, reducing its mineralization and weakening; on the contrary, even moderate but regular physical activity helps maintain bone density. Comorbidities may affect the osteoporosis course and increase the risk of multiple fractures: many diseases or disorders can increase the risk of osteoporosis, directly or indirectly. Moreover, they require the use of specific drugs, which in turn negatively affect the skeleton or imply inactivity or reduced mobility (29). Further, air pollution is associated with decreased bone mass (30).

### 2.4 Epidemiology and etiology

Osteopenia and osteoporosis occur when BMD decreases due to an osteo-metabolic imbalance that disrupts the microarchitecture of bone tissue (31). The third decade of life marks the peak of bone mass, and the exact age varies with gender and skeletal site. After peaking, both sexes undergo a decline in bone mass, and the loss can reach pathological levels, like during menopause in women.

Osteopenia indicates a BMD value lower than the average reference but not low enough to reach the diagnostic criteria of osteoporosis (32). Osteoporosis affects approximately 200 million women worldwide, often resulting in painful fractures (33). A high fracture risk involves about 23 million people in the European Union (34).

Primary osteoporosis forms are related to female post-menopausal state (Type I) due to estrogen deficiency or advancing age (Type II) in men and women. Secondary osteoporosis derives from pre- or co-existing pathologies, medical conditions, or medications interfering with physiological bone formation (35).

The term “fragility fractures” indicates all the fractures resulting from low-level or low-energy trauma (e.g., a fall from standing height or less), which are generally considered the clinical outcome of osteoporosis (36). The most relevant consequence is that a patient may be at high risk of experiencing a secondary fracture in the first 2 years after the initial fracture. The severe complications include increasing morbidity and mortality risks (37, 38).

### 2.5 Diagnosis and therapeutical approaches

According to the World Health Organization’s (WHO) indications, osteopenia diagnosis relies mainly on BMD evaluation by densitometric investigation. The dual-energy x-ray absorptiometry (DEXA) technique at the lumbar spine, proximal femur, or total hip is recognized as a standard diagnostic criterion (39).

However, screening programs with standard DEXA in women over 50 are primarily available in Western countries (40); in men, a diagnosis is usually made only when a fracture occurs.

Other imaging techniques are quantitative computerized tomography, quantitative ultrasound, and conventional radiology (41, 42).

The first approach for treating osteoporosis is correcting or eliminating the “modifiable risk” factors. A dietary intake or supplementation with vitamin D and Ca is usually suggested (43) as a prerequisite for drug treatment.

Current pharmacological therapies pertain to either antiresorptive or osteoanabolic drugs or are dual-acting. Antiresorptive therapies, incrementing bone mass by inhibiting bone resorption, encompass bisphosphonates (the most extensively used osteoporosis drug), monoclonal antibody denosumab, estrogens, and selective estrogen receptor modulators (SERMs). Osteoanabolic therapies aim to increase bone mass by stimulating bone formation. The most frequent approach relies on parathyroid hormone (PTH) activating effects: Teriparatide is a recombinant human PTH and Abaloparatide is a synthetic analog acting like teriparatide. Romosozumab is a monoclonal antibody hindering sclerostin, a significant inhibitor of bone formation (44).

Therapies for osteoporosis can exert several adverse effects, such as gastrointestinal irritation, musculoskeletal discomfort, and bone pain. However, rare but severe adverse effects can occur upon long-term exposure or high doses, like osteonecrosis of the jaw or atypical femoral fractures (45).

## 3 Nutraceuticals for bone health

The concept of health has evolved to emphasize a decreased risk of developing diseases rather than being a illness-free state. This shift has also brought attention to the protective and preventive role of healthy nutrition, conveying confidence in the benefits of nutraceuticals and increasing the demand and supply (46–51).

The term “nutraceutical” is a blend of “nutrient,” which refers to nourishing food, and “pharmaceutical,” which signifies medical drugs. Nutraceuticals offer health benefits that go beyond their traditional nutrients, such as preventing pathological conditions or in addition to conventional drugs (52). On the other hand, dietary supplements are products specifically formulated to enhance the diet by providing a concentrated or extracted form of nutrients (53).

Nutraceuticals are a vast, non-uniform, and ever-expanding group. They can be natural/traditional (directly derived from natural sources) or unnatural/non-traditional (artificially synthesized through agricultural breeding or biotechnology) (53).

**Figure 1:**
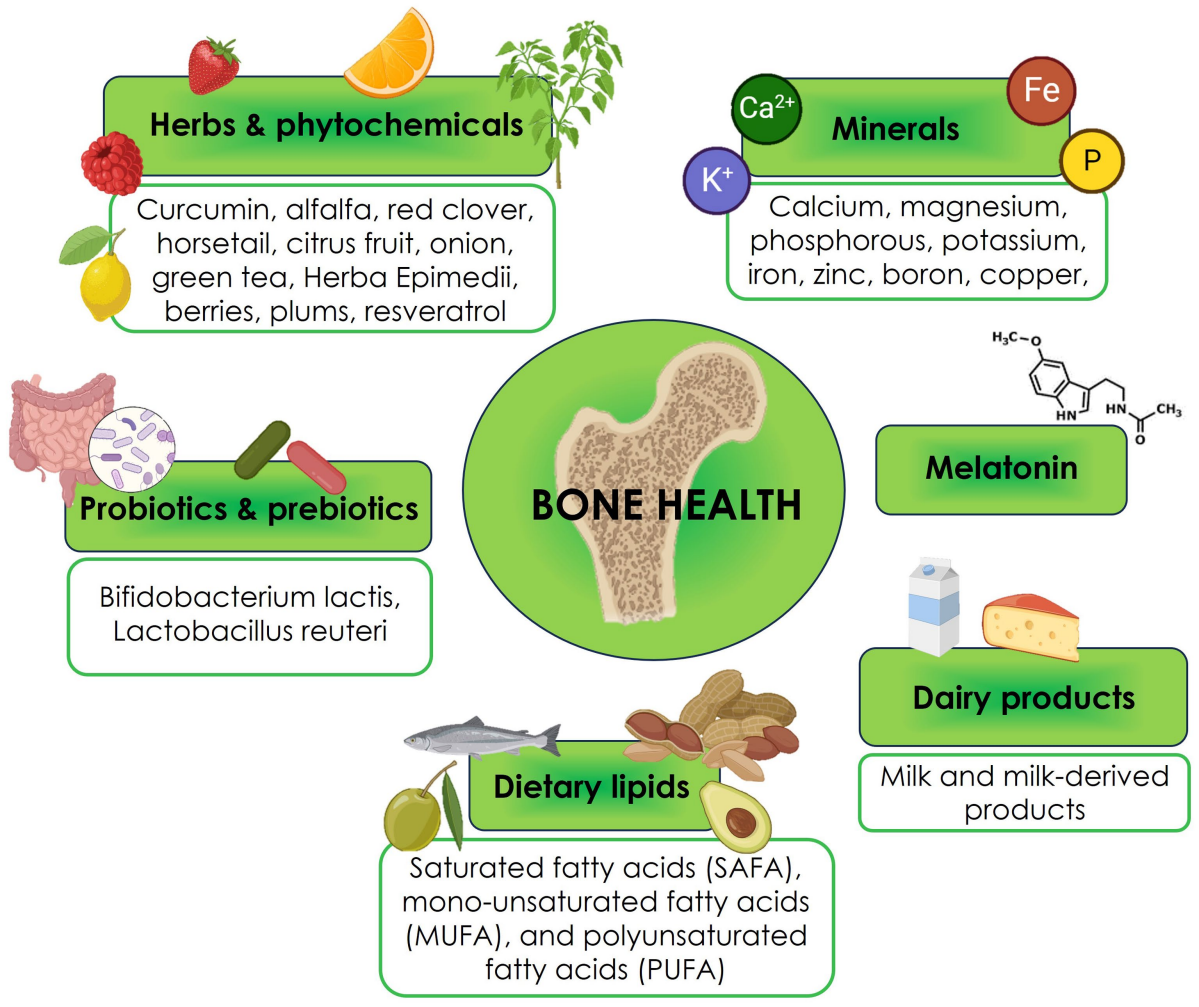
Nutraceuticals for bone health.

Six main nutraceutical groups are beneficial for bone health: minerals, herbs, phytochemicals, dairy products, probiotics and prebiotics, dietary lipids, and melatonin (Figure 1). In the following paragraphs, we will resume the updated existing literature (54).

### 3.1 Minerals

This group includes essential human nutrients, except for boron (B).

#### 3.1.1 Calcium

The 90% of Ca is in bones and teeth as salt. This mineral is crucial for the growth and development of the skeleton, bone mineralization, and achieves peak BMD during adolescence (55). Ca also plays a crucial role in enhancing the effectiveness of bisphosphonates (56). Oxalic and phytic acids in some foods and high sodium consumption can hinder Ca absorption (57).

Ca is key in bone regeneration pathways, including Wingless (Wnt) and β-catenin. Wnts can mediate antagonism of β-catenin signaling through Ca-dependent or independent mechanisms. Additionally, Ca participates in the crosstalk between β-catenin-dependent and independent pathways (58).

#### 3.1.2 .Magnesium

About 60% of the human body’s Mg remains in the bones. Like Ca, it can be mobilized by increased intestinal absorption to maintain serum levels, which is helpful for several body functions. Mg is essential for energy metabolism as it binds ATP and is a cofactor for many enzymes; its positive charge plays a vital role in membrane stabilization. It contrasts the acidic environment of inflammation and protects the skeleton from releasing inflammatory cytokines that stimulate bone loss (59). However, an excessive intake may interfere with the formation of hydroxyapatite crystals due to competition with Ca, consequently reducing bone mass. Conversely, Mg deficiency enhances osteoclast and reduces osteoblast activity, decreases bone stiffness, weakens the apatite crystal structure, and interferes with vitamin D and parathyroid hormone (PTH) and, consequently, Ca homeostasis (60, 61).

Like Ca, Mg can activate the β-catenin pathway and the Wnt signaling of human bone marrow MSCs (BMSCs), promoting bone regeneration. Moreover, by regulating RANK and RANKL, Mg supplementation could prevent bone resorption while promoting bone formation (58).

#### 3.1.3 Phosphorus

Phosphorus (P) is predominantly found in bones and teeth in the hydroxyapatite form (mostly Ca hydroxyapatite), which is crucial for bone mineralization. P can be found as PO_4_^3−^, which regulates bone cell cycle and metabolism and alters signal transduction pathways and gene expression.

High PO_4_^3−^ levels, in concomitance with low Ca intake, induce an increase of PTH, which, in turn, mobilizes Ca from bones (62). Excessive PO_4_^3−^ intake or hyperphosphatemia can lead to ectopic calcification and has been linked to various health issues, including bone disorders. Therefore, maintaining an appropriate PO_4_^3−^ balance in the body is crucial for overall bone health (58).

#### 3.1.4 Potassium

Potassium (K) balances the endogenous acids produced by acid-generating foods like meat, maintaining an alkaline environment. This mechanism helps preserve bone Ca, which is available for mobilization to maintain normal pH. Therefore, consuming foods that are rich in K can help prevent Ca loss from the bone (62).

K-rich diets have been associated with a lower risk of osteoporosis. K-rich foods, such as fruits and vegetables, have an alkalizing effect: bones can neutralize blood acidosis conditions by releasing Ca, potentially reducing bone density. K supplementation improves Ca retention, decreasing bone resorption. High extra-cellular K levels activate the K channels in osteoblasts and osteoclasts, specifically the inwardly rectifying K and voltage-gated K channels. Osteoblasts undergo membrane hyperpolarization, triggering Ca influx and leading toward bone mineralization. On the contrary, osteoclasts are inhibited in their activity (63). Optimal K levels are necessary for collagen production and converting vitamin D to its active form is essential for Ca absorption and bone remodeling (58).

#### 3.1.5 Iron

Iron (Fe) is an enzyme cofactor that helps modulate bone metabolism (62). In most cases, Fe ions are present in the insoluble oxide form that can be changed to a soluble form suitable for body functions. This conversion generates free radicals (64). Excess Fe or reactive oxygen species (ROS) has two effects on bone: they can either activate osteoclast or inhibit osteoblast activity. The first effect occurs through enhanced RANKL activation, suggesting an impact on Wnt signaling. The second is through the mitigation of the differentiation process conducted by bone morphogenic proteins (BMP) and Wnt, which trigger the Runt-related transcription factor 2 (Runx2) and xx (Osx) (58).

#### 3.1.6 Zinc

Zinc (Zn) activates osteoblasts, favoring collagen synthesis and alkaline phosphatase activity. It can influence β-catenin signaling, evidenced by activating the Wnt pathway components Axin2 and LRP5. The osteoclast gene RANKL is also upregulated, suggesting Zn’s ability to regulate osteoclastogenesis (65). At least 20% of people are at risk of Zn deficiency, which correlates to osteoporosis (66).

#### 3.1.7 Boron

Boron (B) is present in high concentrations in bone. It has beneficial effects in combination with Ca, Mg, and vitamin D. It boosts the half-life of vitamin D and estrogen, preserving them and preventing Ca loss and bone demineralization (67). Deficiency of B is associated with osteoporosis, possibly by modulating the Wnt/β-catenin pathway (58).

#### 3.1.8 Copper

Copper (Cu) is involved in collagen fibril formation, angiogenesis, and osteogenic differentiation (62, 68, 69). It is a cofactor for antioxidant enzymes and destroys free radicals (70).

Cu has been found to play a role in activating the β-catenin signaling pathway and in down-regulating Wnt signaling (58).

### 3.2 Herbs and natural phytochemicals

Phytochemicals are chemical bioactive components of nutrient plants that may provide desirable health benefits beyond nutrition. They can reduce the risk of osteoporosis. Phytochemicals include several compounds: terpenoids, polyphenols, alkaloids, organosulfur compounds (OSCs), and phytosterols (71).

#### 3.2.1 Curcumin

*Curcuma longa* commonly known as turmeric, noted for its coloring, flavoring and digestive properties, was originally used as a spice in India.

Curcumin (Cur), a polyphenolic chemical constituent derived from turmeric, can help prevent bone loss by reducing NF-κB, Wnt/β-catenin, RANKL, and tumor necrosis factor-alpha (TNF-α) (72–74), and enhancing the differentiation of osteoblasts from adipose tissue-derived human MSCs through inhibition of Wnt/β-catenin signaling (75).

Cur is a powerful antioxidant that prevents bone weakening by removing harmful molecules and reducing cell death. It achieves this by activating protein kinase B (Akt) to reduce the activity of glycogen synthase kinase-3 beta (Gsk3β), reducing the activity of a critical protective protein, nuclear factor-like 2 (Nrf2) (75).

Cur plays a role in bone resorption by blocking the differentiation of osteoclast precursors into osteoclasts by inhibiting the production of the chemokine CCL3. Cur inhibits osteoclast differentiation markers, including cathepsin K, matrix metalloprotein-9 (MMP-9), and MMP-13, by upregulating miR-365 expression.

Cur exerts immunomodulatory effects on macrophages by inhibiting inflammatory responses, reducing the release of inflammatory factors, and preventing osteoclast formation by improving Akt/NF-κB/NFATc1 signaling (76–81).

#### 3.2.2 Alfalfa

*Medicago sativa* L. is a native plant of Eurasia that contains ipriflavone, which inhibits bone resorption, enhances osteoblast proliferation, induces estrogen-mediated calcitonin secretion, and strengthens estrogen-protecting action on bones (82, 83).

#### 3.2.3 Red clover

Red clover is the extract from *Trifolium pratense* L. It contains genistein and diadzein, phytoestrogens with structural similarity to estrogen, thus preventing bone loss (84, 85).

#### 3.2.4 Equisetum

Also known as horsetail, equisetum is a plant that grows naturally in the northern hemisphere. It contains silica, flavonoids, and triterpenoids, all beneficial for bone health (86, 87).

#### 3.2.5 Citrus fruits

Citrus fruits contain carbohydrates, fiber, minerals, vitamins A, E, and B, and antioxidants such as flavonoids, vitamin C, phenolic compounds, and terpenoids (88).

Compared to other fruits, they are valuable sources of Ca (88–90). Two flavonoids, hesperidin and its aglycone, hesperetin, have protective roles in the osteogenesis of MSCs (90–95). In an ovariectomized mouse model of osteoporosis, hesperetin improved bone volume ratio and bone thickness and decreased trabecular separation (95). Liu et al. (96) also reported the resorption effect of hesperetin. The authors attributed these effects to the elimination of ROS and inhibition of NF-κB and mitogen-activated protein kinase (MAPK) signaling pathways. Further, authors found that hesperetin reduced trabecular bone loss in lipopolysaccharide-induced osteoporotic mice (96).

#### 3.2.6 Alliaceae and Brassicaceae

Naturally derived organic sulfur compounds (OSCs) are molecules containing sulfur, predominantly found in edible plants belonging to the *Allium* and *Brassica*. OSCs promote cell proliferation and viability of MSCs while inhibiting the proliferation and viability of monocytes. OSCs promote osteogenic differentiation and bone formation, inhibit at different stages osteoclast differentiation, reduce bone erosion, and inhibit the viability of osteocytes. Additionally, certain OSCs, such as allicin, allyl sulfide, sulforaphane, and glucoraphanin, are known to modulate bone processes (97).

Polysulfides in Alliaceae, glucosinolates, and isothiocyanates in Brassicaceae have anti-inflammatory, antioxidant, vasorelaxant, and hypolipemic potential. These compounds can induce osteoblast and reduce osteoclast activities by releasing hydrogen sulfide, a gasotransmitter (97).

Flavonoids and organo-sulfur conjugates are present in onion (*Allium cepa* L.), which offers numerous bone health benefits (98), including inhibiting bone resorption (99, 100) and positively modulating BMD in peri- and postmenopausal women (101).

When administered in tablet form over 30 days, garlic (*Allium sativum*) can decrease oxidative stress in menopausal women, which may reduce osteoporosis onset and progression (102). Garlic and leek can also suppress bone resorption (103, 104).

#### 3.2.7 Green tea

Green tea is a beverage derived from dried leaves of *Camellia sinensis* that contains catechins, like epicatechin and epigallocatechin (105). Epigallocatechin—gallate can suppress osteoclast formation and bone resorption by inducing osteoclast cell death, suppressing MMP-9 in osteoblast, inhibiting interleukin-6 (IL-6), suppressing p44/p42 mitogen-activated protein, and down-regulating RANKL-induced expression. At the same time, epigallocatechin-3-gallate can reduce TNF-α and IL-6, promoting osteoblast survival and positively acting on bone formation (99, 104).

#### 3.2.8 Herba Epimedii

Herba Epimedii is a traditional Chinese medicine that contains icariin. This flavonoid glycoside can arrest inflammatory bone loss by inhibiting osteoclastogenesis-induced lipopolysaccharide, TNF-α, and IL-6 and producing type-2 cyclooxygenase and prostaglandin E2 (106).

#### 3.2.9 Berries

Berries contain vitamins A, B9, C, E, and K, minerals, and carotenoids, and are rich in fumaric and citric acid. Berries possess antioxidant (107) and anti-inflammatory properties and can improve osteoblasts differentiation. For example, raspberry trans-retinoid acid and ketones stimulate osteoblast differentiation by improving osteocalcin expression in stem cell cultures.

In addition, berries contain phenolics like flavonoids (anthocyanins, flavonols, and flavanols), phenolic acids, proanthocyanidins, ellagitannins, gallotannins, and stilbenoids. Anthocyanins seem to inhibit RANKL and osteoclastogenesis (108).

#### 3.2.10 Dried plums

Dried plums contain carbohydrates, vitamins A, B, and K, Ca, K, Mg, boron, selenium, dietary fibers, and polyphenols, such as chlorogenic acid, rutin, and proanthocyanidin. Polyphenols decrease bone resorption, acting on RANKL signaling (109). Administering dried plums to postmenopausal females increased insulin-like growth factor 1 (IGF-1) and bone-specific alkaline phosphatase (BSAP) levels (110).

#### 3.2.11 Resveratrol

Resveratrol (RSV) is a polyphenol in red wine, nuts, grapes, and cranberries.

RSV functions as both anabolic and antiresorptive agent. Indeed, RSV is a SIRT1 activator or directly acts on Runx2 and Osx to regulate osteoblast differentiation; it also activates Runx2 and OSX expressions, RANKL/OPG, NFACT1, and NF-kB expressions to regulate osteoclast differentiation. In addition, it displays antioxidant and anti-inflammatory features (111, 112).

These dual actions of RSV on osteoblast and osteoclast are beneficial in maintaining balance in bone remodeling, a critical endpoint in the management of osteoporosis (113).

### 3.3 Dairy products

Milk and milk-derived products contain Ca, lipids, proteins, K, sodium, Zn, P, and vitamins A and B2, valuable substances for bone loss prevention. Functional milk contains compounds like casein phosphopeptide, milk basic protein (MBP), and lactoferrin, enhancing osteoblast proliferation and differentiation while preventing osteoclast formation (114–116). Oliveira et al. studied the effect of drinking water supplemented with milk extracellular vesicles (mEVs) on mice with diet-induced obesity and on OVX mice. Mice from both models showed a systemic and local decrease in the RANKL/OPG and were protected from bone loss and osteoclastogenesis. *In vitro* experiments corroborated those results (117).

Fermented dairy products, including yogurt, soft cheese, and Kefir, positively influence bone growth and homeostasis through different mechanisms involving intake of essential nutrients such as Ca P, protein, and potentially pre-and probiotics (118). Kefir is formed when milk is fermented by lactic acid bacteria and yeasts enclosed in a protein-and-polysaccharide matrix. Fermentation can negatively affect IGF-I due to lactic acid bacteria, which can utilize IGF-I or IGF-binding protein complex as their nutrition source (119). Dahiya et al. observed that kefir supplementation in OVX mice caused increased BMD (120).

Cheese is an excellent source of Ca, vitamin D, and high-quality proteins such as casein and whey, which contain all the essential amino acids except methionine and cysteine (121). These nutrients are essential for bone structure and maintenance. Parmigiano Reggiano cheese is considered a “functional food” for bone health and osteoporosis prevention due to its high-biological value proteins and easily accessible Ca (122). Swiss cheese contains H_3_PO_4_ and protein in large amounts. Gouda and Brie cheeses contain vitamin K2, which is associated with improved Ca metabolism and acts as a cofactor in glutamic acid carboxylation, essential for controlling bone metabolism (121).

### 3.4 Probiotics and prebiotics

Gut microbiota (GM) is a complex microflora consisting of millions of microbes in humans. Under physiological conditions, GM lives in a balanced state known as eubiosis, forming multidirectional connections with other organs such as the brain, gut, and bone axis while interacting with many pathways (123, 124).

Changes in GM can cause dysbiosis, impacting on bone remodeling and metabolism (125). This process can occur directly through extracellular vesicles (EVs), short-chain fatty acids (SCFAs), polyamines, and hydrogen sulfide or indirectly by interacting with immune cells or hormones (126). The GM may regulate bone metabolism by interacting with the Wnt/β-catenin signaling pathway, which relies on Ca. Differences in GM compositions have been detected in women with normal BMD, osteopenia, or osteoporosis (127).

#### 3.4.1 Probiotics

Probiotics are beneficial bacteria present in GM (128, 129), such as *Lactobacillus*, *Bifidobacterium*, *Escherichia coli*, *Enterococcus*, *Bacillus subtilis*, and *Saccharomyces* species. By impacting the RANKL/RANK/OPG pathway, they can favor bone anabolism and decrease bone loss caused by estrogen deficiency (130). *Lactobacillus reuteri* administration prevented type-1 diabetes-induced osteoporosis by inhibiting TNF-mediated suppression of Wnt10b. It enhanced bone density by increasing osteoclast activity or restoring Wnt10b suppression in mice with glucocorticoid-induced osteoporosis. Wnt10b can increase osteoblastogenesis by downregulating the expression of PPAR-γ (124, 127).

#### 3.4.2 Prebiotics

Prebiotics are non-digestible food ingredients that can promote bacteria growth and activity by acting as a substrate of selective fermentation from the intestinal microflora. In most cases, they are products of the enzymatic conversion of sugars. Among them, non-digestible oligosaccharides (NDOs) like lactulose, galactooligosaccharides (GOS), fructooligosaccharides (FOS), oligofructose, inulin, lactose derivatives, xylooligosaccharides (XOSs), and soluble corn fiber (SCF) can enhance mineral absorption and reduce bone resorption. Resistant starches are a dietary fiber subgroup promoting soya isoflavone production and increasing the ratio of *Bifidobacteria*, *Lactobacillus* species, and *Bacteroides*. These starches reduce bone loss in OVX mice, decrease inflammation, and interfere with the RANKL/OPG pathway. The prebiotics share the GM’s ability to convert to SCFAs, including acetate, propionate, and butyrate. They can affect Ca and Mg intestinal absorption in animals and humans. The process occurs by lowering the cecal pH and BMD (123, 131–135).

#### 3.4.3 Synbiotics

Synbiotics combine probiotics and prebiotics in synergy and enhance their effects and characteristics. In OVX rats, FOS and soy isoflavone mixture increased bone trabecular microarchitectural properties (136); FOS combined with dried plum fraction in soy-based diets resulted in greater whole-body BMD (137). FOS and *Bifidobacterium longum* increased the Ca, Mg, and P content of bone and bone-breaking force in rats (138). Further, combining *Bifidobacterium longum*, and GOS augmented Ca, Mg, and P bioavailability and hind limb bone mineral content (139, 140).

#### 3.4.4 Postbiotics

Postbiotics have recently been defined as bioactive compounds produced by food-grade microorganisms during fermentation (141). Postbiotics is an umbrella term that includes metabolites, SCFAs, microbial cell fractions, functional proteins, extracellular polymeric substances (EPSs), cell lysates, teichoic acid, peptidoglycan-derived muropeptides, and pili-type structures, which may be used to promote health by modulating the GM. Only a few recent studies have shown how postbiotic administration ameliorates osteopenia and osteoporosis (142). In OVX rats, the *Bacillus coagulans*-derived-postbiotics positively impacted on BMD (143). In a murine model of postmenopausal-related osteoporosis, the postbiotic *Lactobacillus curvatus* 38-CS affected RANKL-induced osteoclast differentiation and bone loss by downregulating the TRAF6/NF-κB/MAPKs axis (144).

### 3.5 Dietary lipids

Dietary lipids enclose saturated (SAFAs), monounsaturated (MUFAs), and polyunsaturated (PUFAs) fatty acids based on the double bond number and common chain length.

#### 3.5.1 SAFA

Animal-based food is the primary source of nutrition for SAFAs.

High-fat diets (HFD), particularly those rich in saturated fatty acids (SAFA-HFD), can harm bone health. These diets can lead to an imbalance between bone resorption and formation, as well as an increase in DNA damage, potentially contributing to bone loss (145).

Oxidative damage is responsible for high osteocyte apoptosis in SAFA-HFD-fed animals compared to those fed a standard diet; micropetrosis and the canalicular system disruption cause this, leading to bone fragility. Moreover, SAFA-HFD strongly inhibits osteoclast apoptosis in IL-6-deficient mouse models. Consequently, survival of osteoclasts and apoptosis of bone-forming cells may lead to osteoporosis (145).

#### 3.5.2 MUFA

MUFAs are abundant in nuts, avocados, olive oil, and canola (rapeseed). Olive oil, one of the essential foods in the Mediterranean diet, contains MUFA in the form of oleic acid. In extra virgin olive oil (EVOO), phenolic compounds are also present, which have a beneficial role. Fat from meat also contains MUFAs and SAFAs but is not recommended as a good source of MUFAs (146).

#### 3.5.3 PUFA

PUFAs enclose the omega-3 (*n*-3) mainly derived from α-linolenic acid (ALA) and omega-6 (*n*-6) groups, primarily synthesized from linoleic acid (LA) (147). Humans do not synthesize ALA and LA, so all the sources are exogenous (148). The *n*-6 PUFAs are in vegetable oils such as sunflower, corn, cottonseed, and safflower. In contrast, the *n*-3 PUFAs, such as eicosapentaenoic (EPA) and docosahexaenoic (DHA) acids are found in fish and fish oil. PUFAs are beneficial for bone health, primarily ALA: *n*-3 PUFAs supplementation decreases bone resorption (measured as CTX marker) and increases BMD (149).

To enhance bone health in advanced age, dietary oils rich in the *n*-6 fatty acid gamma-linolenic acid (GLA) can be combined with the *n*-3 PUFA EPA to reduce PGE2 synthesis and enhance PGE1 production, which has anti-inflammatory effects. Decreasing the amount of *n*-6 PUFAs allows the manipulation of endogenous prostaglandin synthesis (145).

In animals fed with PUFA-rich diets, *n*-6 and *n*-3 PUFA may interact with PPAR-γ to inhibit osteoblast differentiation and promote the adipocyte one (145).

Animals fed with MUFA-rich diets had superior BMD values to those fed *n*-6 PUFA-rich diets. However, PUFA-rich diets were more beneficial to bone than SAFA-rich diets (145).

PUFA or HFD-SAFA-rich diets increase ROS production in growing mice compared to other unsaturated fat alimentations. This activates osteoclasts and inhibits osteoblast maturation through NF-κB, enhancing adipogenesis (145).

### 3.6 Melatonin

Melatonin (Mel) is a bioamine (N-acetyl-5-methoxytryptamine) secreted and released by the pineal gland at night. It controls circadian rhythms, body temperature, reproduction, immune and cardiovascular systems, energy, and bone metabolism. Mel predominantly acts through its cognate receptor, Mel receptor 2 (MT2R), expressed on MSCs, osteoblasts, and osteoclasts (150).

Mel has been shown to have an anti-osteoporotic effect since it induces beneficial effects on bone tissue. Altered levels of Mel are associated with the occurrence and development of osteoporosis in postmenopausal women (151).

Mel is an inducer of osteoblast proliferation and differentiation, mineralized ECM formation, and cortical bone formation (152). In Wistar rats, it improved bone mass, volume, and stiffness and increased skeletal strength compared to the control group (153). Mel promotes osteogenic differentiation by multiple mechanisms, including receptor-mediated signaling to stimulate osteogenic genes, support BMP signaling, upregulate OPG, and downregulate PPARγ expression. These effects are achieved by MT2R-dependent signaling involving the classical signaling molecules and antioxidant effects of the hormone and RNAs. As a result, Mel stimulates osteoblast formation and downregulates osteoclast and adipocyte formation (150, 151). In a rat model, Mel inverted the decrease in the osteogenic differentiation ability of bone marrow MSCs, a condition usually associated with osteoporosis. Additionally, it reversed TNF-’s anti-osteogenic differentiation and inflammation in BMSCs by suppressing the NF-kB signaling pathways (154).

Mel can enhance the osteogenic differentiation of BMSCs and retards bone loss through the HGF/PTEN/Wnt/β-catenin axis in OVX mice. Additionally, HGF diminished the expression of PTEN, resulting in an activated Wnt/β-catenin pathway both *in vitro* and *in vivo* (155).

Mel promoted BMSC-mediated osteogenesis-angiogenesis coupling in OVX rats with tibia defects as an osteoporotic model. Mel also promoted angiogenesis by upregulating VEGF levels and enhanced the expression of the typical osteogenesis and angiogenesis-related markers compared to the untreated group (156).

Mel suppressed the osteoclastogenic RANKL and upregulated the anti-osteoclastogenic RANKL decoy receptor OPG in osteoblasts, exerting an anti-resorptive effect. Thus, Mel antagonizes the osteoclastogenic function, also inhibiting the SIRT signaling that acts as a suppressor of the NF-κB signaling pathway that, on the contrary, leads to enhanced osteoclastogenesis. In addition, Mel inhibits the osteoclastogenic differentiation of hematopoietic stem cells and protects bone marrow cells exposed to a cytotoxic drug, aracytin. However, the cellular source of Mel in bone marrow is unknown (157).

In a rat model for glucocorticoid induced osteoporosis, high doses of dexamethasone caused BMSCs to undergo ferroptosis, a programmed cell death that depends on Fe. The prevention of osteoporosis was demonstrated by early Mel inhibition of the ferroptosis pathway. Indeed, Mel was able to block the phosphatidylinositol 3-kinase (PI3K), protein kinase B (AKT), and mammalian target of the rapamycin (mTOR) axis that controls bone metabolism (158).

In the type 2 diabetic osteoporotic mouse model, high glucose triggers ferroptosis by increasing ROS, lipid peroxidation, and glutathione depletion. However, administration of Mel can inhibit ferroptosis by triggering the nuclear factor erythroid 2-related factor 2 (Nrf2) and heme oxygenase-1 (HO-1) signaling pathway. This helps improve the osteogenic capacity of osteoblasts. These findings have been confirmed through *in vitro* experiments (159).

In osteoclasts, Mel inhibits differentiation and function by suppressing RANKL-induced ROS production by inhibiting NF-κB activation. It attenuates the damage induced by oxidative stress and inflammation on osteoblasts and prevents osteolysis from ROS and inflammatory factors. Mitochondria is the central organelle that generates free radicals and oxidative stress, contributing to aging-related diseases, including osteoporosis. Moreover, mitochondrial dysfunction inhibits osteogenesis and favors osteoclastic function, contributing to bone loss during aging. Mel could serve as an endogenous mitochondria-targeted antioxidant to diminish oxidative stress in bone cells more efficiently, thereby preventing bone loss (160).

## 4 Legislation framework

An internationally recognized definition of nutraceuticals is absent and, consequently, different definitions have emerged over the years, generating confusion. Nutraceuticals are regulated as food category in some countries, focusing on safety and labeling (161). However, they do not always have a specific definition, and, in some countries, supplements, herbal products, pre-and pro-biotics, and functional and fortified foods are not classified as nutraceuticals (Table 1).

**Table 1:** Overview of regulation for nutraceuticals.

Area/Country	Regulation	Ref/Link
European Union	The European Food and Safety Authority (EFSA) regulates food legislation and considers food as supplement foods	EU regulation CE n. 178/2002
	The European Commission has harmonized rules to safeguard consumers against possible health risks from ingredients different from vitamins and minerals	http://www.efsa/eu
	New products must meet strict development and quality requirements. Before they reach the market, EFSA must authorize any health claim, and then each EU State can set a specific approval regulation. However, EFSA is not mandated to act against unsafe products once they are on the market	Directive 2002/46/EC
United States	Foods, drugs, and nutraceuticals are regulated separately, according to the Food and Drug Administration (FDA). FDA does not need to approve or register nutraceuticals before producing or selling them. However, nutraceuticals must meet high standards of trial design and patient safety and be reviewed by the Investigational New Drug (IND) application process. FDA can act against an unsafe product only after it is on the market. Manufacturers must ensure nutraceutical safety and efficacy before market release	(208); Dietary Supplement Health and Education Act (DSHEA)
India	Nutraceuticals have no specific legal status	Food Safety and Standard Act, 2006
Australia	Nutraceuticals come under the food category and, therefore, fall into the national regulations for food	Therapeutic Goods Administration (TGA)
Canada	Supplements and nutraceuticals are regulated more closely as drugs than food categories, and there are explicit rules for them	(209, 210)
Latin America	Colombia, Brazil, and Argentina require the registration of a new nutraceutical in the market. In Brazil, animal and human clinical studies are mandatory before product registration. Mexico and Chile follow a notification-based approach	(208)
China and Taiwan	Before product registration, regulators require clinical studies on animals and humans	(208)
Japan	The first country to regulate functional foods through the establishment of Foods for Specified Health Use (FOSHU) in 1991. Later, this evolved into the 2003 Health Promotion Law, which allowed foods with beneficial health activities and meeting FOSHU requirements to obtain approval as nutraceuticals. Japan has a well-established market in nutraceuticals dating back to the 1980s	(161)

For a detailed update on the regulatory framework and policies concerning nutraceuticals in the global markets, see Chopra’s et al. (162) review.

Nutraceuticals require a sharp classification and shared regulation to protect the category, safeguard consumers’ health, and avoid counterfeits.

## 5 Clinical trials

Clinical trials are crucial for understanding the dosage, mechanism of action, and side effects of nutraceuticals in managing osteoporosis. Therefore, we conducted a literature research through the PubMed database, with the CLINICAL TRIAL filter and the keywords “type/name of the nutraceutical,” “osteopenia,” “osteoporosis,” “bone loss,” and “bone fragility.” We considered papers published from 2000 until today and written in English. We excluded one paper written in Chinese and one in Russian. We also evaluated previous reviews on this topic.

The trials we found did not cover all nutraceuticals, and the data obtained so far needs to be consolidated. Our research led us to diverse studies, revealing homogeneities and heterogeneities (Supplementary Tables S1–S6). We found some homogeneity in the chosen population, such as gender (female), age (advancing years), and hormonal status (post-menopausal). On the other hand, we encountered much heterogeneity due to variations in sample sizes, methodologies, doses, administration lengths, outcomes, adverse effects, and follow-up (163–177). We found three studies on dosage effects (178–180).

A study evaluating Ca supplementation’s long-term effects (5 years) revealed that the bone benefits did not persist once supplements were stopped (181). Men with primary osteoporosis administered vitamin D or Ca supplements reported transient beneficial effects (182). Those studies highlight the need for further research on the long-term effects of nutraceuticals, which could significantly impact osteoporosis management.

Measuring the effects of nutraceuticals on bone health has been challenging, leading various clinical trials to utilize the ^41^Ca methodology. The reason for choosing this approach was that traditional analytical methods were often unable to detect the small effects of those compounds on bone (166, 183).

Some studies we found on probiotics showed beneficial effects on bone, as confirmed by other reviews (149). However, according to the systematic review by Billington et al., some RCTs had a high risk of bias based on the Cochrane Risk of Bias 2 Tool. Furthermore, the effects of probiotics were inconsistent (184). We also found studies evaluating *n*-3 fatty acids but reporting no significant beneficial effects on bone health (185–187).

The side effect issue still needs to be addressed. Some of the reported publications did not indicate any side effects, but it is unclear if they were not evaluated or if there were none.

Finally, we must acknowledge that a few authors question the practicality and feasibility of using randomized clinical trials (RCTs) when studying nutraceuticals (188–194). The multifunctional nature of nutraceuticals can make it hard to estimate their effects and choose the appropriate time points and biomarkers (195). Patients enrolled in a clinical trial for nutraceuticals must maintain specific healthy lifestyle habits and diet regimen not influencing data; this, in turn, can generate a high dropout rate. Another concern is the statistical analysis method generally used in RCTs, which may require some adjustments (196). Some nutrition and nutraceutical studies have employed the N-of-1 study design, which may address their heterogeneity and crossover features, including carry-over effects (197–201).

## 6 Discussion

**Figure 2:**
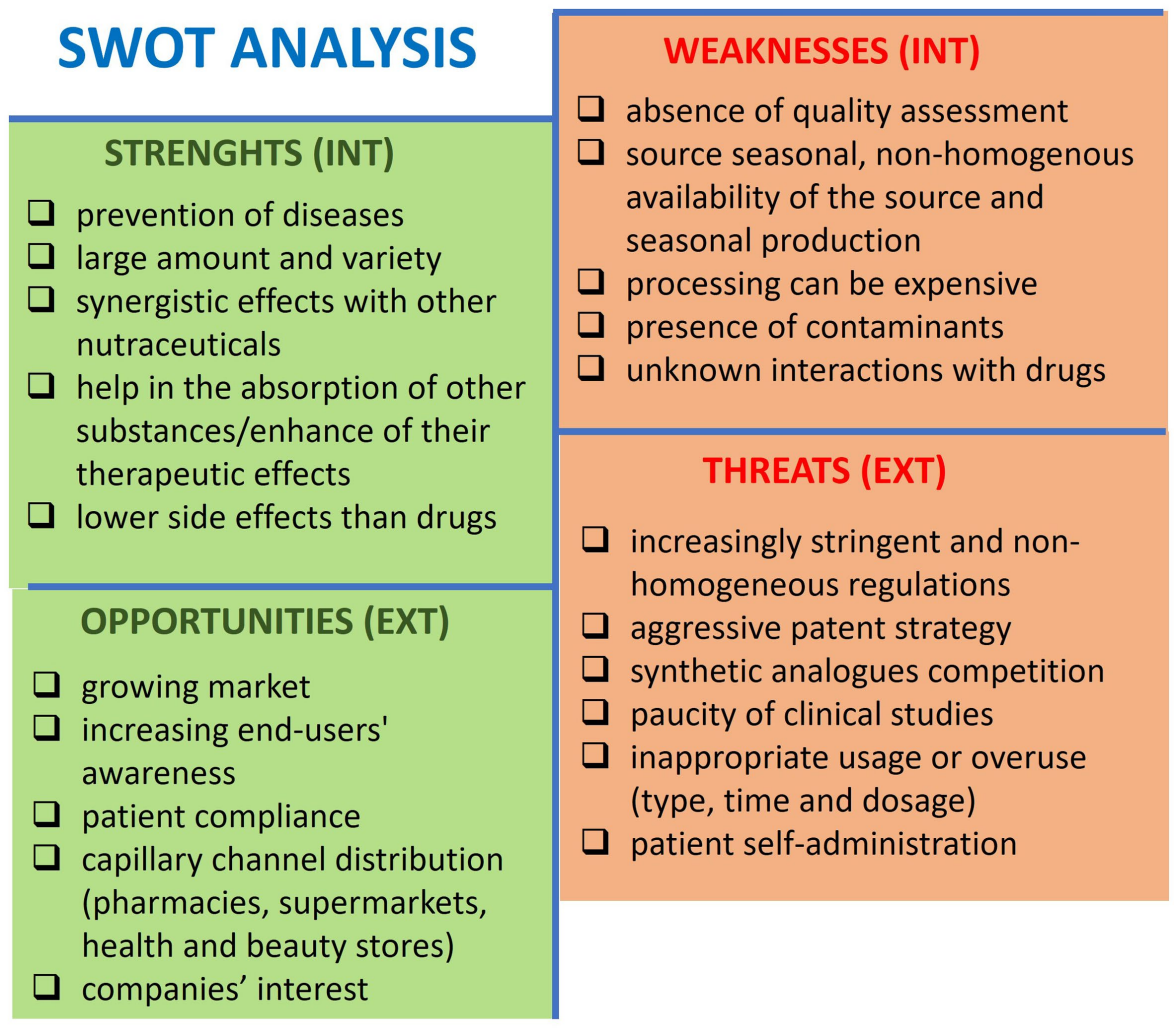
SWOT analysis showing the critical aspects of nutraceuticals for preventing bone loss and osteoporosis. INT, internal context; EXT, external context.

We have utilized the “strengths, weaknesses, opportunities, threats (SWOT)” analysis to identify and discuss the advantages and disadvantages of nutraceuticals in preventing bone loss and osteoporosis, consider internal and external variables, and define goals to achieve and future directions (Figure 2). Strengths are internal factors that can be exploited, whereas weaknesses are internal limitations that must be addressed. Opportunities are external possibilities that can be capitalized on, while threats are external issues that need to be evaluated and faced.

### 6.1 Strengths

Nutraceuticals exist in various forms, such as tablets, capsules, liquids, and powders, which make them easy to consume. They have few side effects (202), becoming promising for prevention. They can also be combined with other nutraceuticals, supplements, or drugs to improve or enhance their therapeutic effects or absorption (54, 203).

### 6.2 Weakness

Nutraceuticals may be available in different countries, considering seasonal variations and crop yield fluctuations, leading to potential supply shortages or price fluctuations. Moreover, the manufacturing process can be complex, time-consuming, and expensive, and it can carry contaminants. Indeed, nutraceuticals can become contaminated during any production stage with drugs, pesticides, mycotoxins, heavy metals, metalloids, and radioactivity. This can lead to declining quality standards and safety; informing the population and healthcare professionals about such issues is essential (204). Consolidating data on the potential interference of nutraceuticals with other drugs is necessary to avoid undesired changes and side effects in absorption, bioavailability, transport, metabolism, and elimination (205).

### 6.3 Opportunities

The global nutraceuticals market is constantly increasing, and academia and sector companies are allocating more resources to Research & Development. The diversity of nutraceuticals allows for a wide distribution range, including hypermarkets, supermarkets, pharmacies, and online sales channels. Consumers are more aware of the potential of natural-based alternatives and personalized nutrition that they prefer over synthetic pharmaceutics, which are perceived to carry side effects.

### 6.4 Threats

The regulation of nutraceuticals varies across different countries and is not consistent worldwide. Differently from drugs, nutraceuticals are subject to less strict production requirements, which can lead to a lack of safety and effectiveness data (206).

There is no proper knowledge about the dosage and intake regimen to avoid inappropriate use or overuse, which can lead to toxic effects and health complications (202, 207).

Natural ingredients that are beneficial to health cannot be patented.

Furthermore, synthetic food supplements such as vitamins, minerals, amino acids, and antioxidants pose significant competition.

## 7 Conclusions and perspectives

Along with behavioral habits, nutraceuticals can help prevent osteoporosis and promote overall well-being in individual with increased risk for severe illness. This potential warrants further research and exploration in nutrition and osteoporosis management.

Our intent to stay abreast of ongoing investigations led us to the ClinicalTrials.gov website (February 2nd, 2024) with the RECRUITING filter. Our search, using keywords such as nutraceutical name (in the field: other terms), bone loss, bone fragility, osteopenia, and osteoporosis (in the field: condition/disease), revealed that while some efforts are underway, there is still a significant gap to bridge (Table 2). The need for more comprehensive and evidence-based data on their mechanisms of action, effects, safety, appropriate dosage, potential adverse effects, and interactions with other compounds is paramount. Defining the safety profile is crucial since nutraceuticals are not subject to reporting adverse reactions to competent bodies.

**Table 2:** Current clinical trials on the effects of Nutraceuticals on bone loss.

Nutraceutical	NCT number	Title
Iron	NCT05489952	Iron supplementation for geriatric hip fractures
Probiotic & prebiotic	NCT05332626	*Lactobacillus acidophilus* and postmenopausal osteoporosis women
	NCT05348694	OsteoPreP: food supplements for postmenopausal bone health
	NCT05009875	Food trial evaluating the efficacy of SBD111 versus placebo for the clinical dietary management of the metabolic processes of osteopenia
	NCT05421819	Design and development of a novel food supplement for osteoporosis based on gut microbiome mechanisms
Melatonin	NCT04233112	Melatonin and osteogenic loading on osteopenia—active, not recruiting
Vitamin D	NCT05421819	Design and development of a novel food supplement for osteoporosis based on gut microbiome mechanisms
