# Supplementary material for: Nutraceuticals in osteoporosis prevention
Source: Front Nutr. 2024 Oct 2;11:1445955. doi: 10.3389/fnut.2024.1445955 (PMC11479890; doi:10.3389/fnut.2024.1445955)
Supplement: Supplementary file 1 [file Table_1.docx]

Supplementary Material

# Supplementary Tables

**Table 1**. Clinical trials on the effects of Minerals on bone loss. Abbreviations: ALP: alkaline phosphatase; BAP: bone alkaline phosphatase; BMC: bone mass content; CTX= carboxy-terminal telopeptide of type I collagen; DEXA: Dual Energy X-ray Absorptiometry; HR-pQCT: high-resolution peripheral; ICP-MS: inductively coupled plasma mass spectrometry; mEq: milliequivalent; NI: Not Indicated; OC: osteocalcin; OPG: osteoprotegerin; PINP: procollagen type 1 N terminal propeptide; Pyd: pyridinoline; QCT: quantitative computed tomography; TEAC: total antioxidant capacity; TRAP5b: tartrate-resistant acid phosphatase isoform 5b; uNTx: type I collagen cross-linked N-telopeptide

| **Nutraceutical form** | **Dose** | **Treatment length** | **Population** | **Population: age (years)** | **Status** | **Type of trial** | **Control group** | **Evaluations performed** | **Results** | **Ref.** |
| --- | --- | --- | --- | --- | --- | --- | --- | --- | --- | --- |
| K citrate | 30 mEq daily | 1 year | 161 postmenopausal women | 58.6 ± 4.8 | osteopenia | Randomized, prospective, controlled, double-blind | 30 mEq K chloride (KCl) | BMD evaluation | increased BMD in the treatment group | (1) |
| K-citrate | 40 mEq + 630 mg Ca citrate and 400 IU vitamin D3 daily | 2 years | 83 postmenopausal women | NI | osteopenia | RCT, double-blind | placebo | bone turnover and bone formation markers, changes in BMD | significant decrease in turnover markers; absence of consistent effect on bone formation markers; no effects on BMD | (2) |
| K citrate, 2 tablet | 30 mEq + Ca carbonate (500 mg) and vitamin D (400 IU) daily | 6 months | 40 >5 years postmenopausal women | NI | osteopenic women with K and citrate deficit and low-grade acidosis | RCT, double-blind | placebo | BMD evaluation, measures of TRACP5b, (CTX), BAP, PINP, and urine pH, electrolytes, and citrate | K supplementation improved the beneficial effects of Ca and vitamin D | (3) |
| Mg citrate | 1,830 mg daily | 30 months | 20 postmenopausal women | 52.7±5.8 Mg group, 4±5.1 control group | osteoporosis | RCT, double-blind | 0 mg | Mg, Ca, P, iPTH, and OC in blood samples. Deoxypyridinoline levels adjusted for creatinine measured in urine samples. | increase serum iPTH and OC levels; decrease deoxypyridinoline levels in urine; suppression of bone turnover | (4) |
| Ca tablets, 3 | 1800 mg Ca/d as triCa phosphate (plus 20 µg teriparatide/d and 1000 IU vitamin D3/d) daily | 1 year | 211 postmenopausal women | 60-85 | osteoporosis | randomized, positive-comparator, 2-arm, single-blind | second arm: 1800 mg Ca/d as Ca carbonate (plus 20 µg teriparatide/d and 1000 IU vitamin D3/d) | variations in lumbar spine and hip BMD; changes in bone resorption markers and serum and urine Ca and P concentrations | no signiﬁcant difference between-group in serum Ca and P concentrations or urine Ca concentrations; bone resorption markers increased in both groups, as expected with teriparatide, but not significatively | (5) |
| Ca, not indicated | 1 g Ca citrate daily | 5 years | 1471 postmenopausal women >5 years | >55 | a normal lumbar spine | RCT, double-blind | placebo | BMD measurements at 10 years | no effect on BMD, total fracture, or hip fracture incidence but significant reductions in forearm and vertebral fractures | (6) |
| Ca pills | 5g hydrolyzed collagen Ca chelate (contains 500 mg of elemental Ca and 200 IU of vitamin D) daily | 1 year | 112 postmenopausal women > 1-10 years | 55.7 ± 3.3 | osteopenia | RCT, double-blind | placebo | whole body BMD; levels of sclerostin and TRAP5b and bone-specific alkaline phosphatase/TRAP5b ratio | attenuation of bone loss; reduced levels of sclerostin and TRAP5b, higher levels of BAP/TRAP5b ratio compared to the control | (7) |
| Ca capsule | 500 mg of ossein-hydroxyapatite (OHC) vs 500 mg of triCa phosphate (TCP) daily | 6 months | 153 postmenopausal women | ≥60 | osteopenia with low Ca intake | RCT double-blind | placebo | OC, PINP, BMD | ossein-hydroxyapatite increased bone density by 0.8% at the spine at 12 months | (8) |
| Ca tablet | 0.25 µg calcitriol twice daily or 500 mg Ca twice daily | 2 years | 41 males | 27-67 | primary osteoporosis | RCT, double masked | double placebo | bone turnover markers, BMD | efficacy of calcitriol alone remains unproven for the treatment of osteoporosis in men | (9) |
| Ca tablet | 600 mg twice daily | 5 years | 1460 women | ≥70, mean 75 | relatively healthy, vitamin D–sufficient | RCT, double blind | placebo | incidence of osteoporotic fractures, vertebral deformity, and adverse events, the bone structure measured by DEXA, quantitative ultrasonography of the heel, and peripheral quantitative computed tomography of the distal radius | supplementation with Ca carbonate tablets is ineffective in preventing clinical fractures | (10) |
| Ca tablet | 830 mg ossein-hydroxyapatite complex (OHC)(712 mg elemental Ca) twice daily | 3 years | 120 women | ≥65 | senile osteoporosis | randomized, open-label, parallel-group, controlled, prospective study | open-label Ca carbonate (500 mg of elemental Ca) twice | BMD, bone remodeling markers | OHC had a greater anabolic effect on bone than Ca carbonate | (11) |
| Zn supplement | 600 mg Ca + 2 mg Cu + 12mg Zn daily | 2 years | 224; postmenopausal women | 51-80 | healthy | RCT, double-blind | placebo (600mg Ca plus maize starch) | BMD, mineral content, T-score | Zn, but no Cu, influenced whole-body BMD, mineral content, and T score | (12) |
| Zn sulphate capsule | 220 mg (containing 50 mg zinc) daily | 1 month | 60; postmenopausal women | 48-89 | osteoporosis | RCT, double-blind | placebo | Serum Zn and Ca levels | the intervention group showed a significantly higher serum zinc concentration than at baseline but no significant difference in serum Ca concentration. No significant differences were found in Ca and zinc concentrations in the placebo group before and after the intervention. | (13) |

**Table 2**. Clinical trials on the effects of Herbs and Phytochemicals on bone loss.

| **Nutraceutical form** | **Dose** | **Treatment length** | **Population** | **Population: age (years)** | **Status** | **Type of trial** | **Control group** | **Evaluations performed** | **Results** | **Ref** |
| --- | --- | --- | --- | --- | --- | --- | --- | --- | --- | --- |
| dried plums | 100 g daily | 1 year | 55 women for dried plums and 45women for dried apples | 57,72 ± 4.08 (dried plums) 55,43 ± 5,18 (dried apples) | osteopenic | RCT | 75 g dried apple | measurement of serum levels of RANKL, OPG and sclerostin | dried plums positively affect bone, suppress RANKL, promote OPG, and inhibit sclerostin | (14) |
| dried plums | 50 g vs 100 g daily | 6 months | 48 postmenopausal women | 65-79 | osteopenic | RCT | no treatments | bone mass, bone-specific alkaline phosphatase (BAP), tartrate-resistant acid phosphatase (TRAP-5b), high-sensitivity C-reactive protein (hs-CRP), insulin-like growth factor-1 (IGF-1), and sclerostin, OPG (OPG), receptor activator of nuclear factor kappa-B ligand (RANKL), Ca, phosphorous, and vitamin D were measured | potential prevention of bone loss | (15) |
| garlic, 2 tablets | 400 mg raw garlic powder (more than 1200 micrograms allicin) | 1 year | 44 postmenopausal women | 45-65 | osteoporotic | RCT, double-blind | placebo | serum IL-1, IL-6, and TNF-α were measured with ELISA assay before and after the intervention. | no significant difference in IL -1 and -6; TNF-α showed no significative difference between the two groups but was significantly reduced by about 47% in the GG group | (16) |
| resveratrol capsules | 75 mg twice daily | 2 years | 62 women in the placebo group and 62 in the resveratrol group | 45-85 | healthy | RCT, double-blind, two-period crossover intervention | placebo group with placebo capsules | effects of resveratrol supplementation on cognitive performance, cerebrovascular function, bone health, cardiometabolic markers, and well-being in postmenopausal women | positive effects in women with poor bone health biomarker status on bone density (increased BMD) with reduced CTX level compared with placebo. Thus, the 10-year probability of hip fracture risk has decreased. The benefit of resveratrol was more remarkable in participants supplemented with vitamin D plus Ca | (17) |
| resveratrol tablet | 200 mg of fermented soy (including 80 mg of isoflavone aglycones and 10 mg of equol) and 25 mg of resveratrol from Vitis vinifera daily | 1 year | 60 menopausal women | 50-55 | healthy | RCT, double-blind, | placebo-controlled dietary intervention | whole-body BMD and bone turnover biomarkers, such as deoxypyridinoline (DPD), tartrate-resistant acid phosphatase 5b (TRACP-5b), OC, and bone-specific alkaline phosphatase (BAP), were measured. | DPD, OC, and BAP significantly improved in the active group, while TRACP-5b levels were unaffected. Overall, the intervention significantly increased BMD measured in the whole body compared with the placebo. | (18) |
| blueberry, freeze-dried powder | 17.5 g vs 35 g vs 70 g daily | 47 months | 14 postmenopausal women | 45-70 | healthy | randomized crossover | no treatment | ^41^Ca methodology, bone resorption serum biomarkers and urinary polyphenols were measured | net bone Ca retention increased by 6% and 4% with the low and medium doses, respectively | (19) |
| blackcurrant capsules | 392 mg (one capsule) vs784 mg (two capsules), daily | 6 months | 40 peri- and early postmenopausal women | 45-60 | healthy | RCT, pilot double-blind | placebo | BMD and bone formation markers | blackcurrant supplementation decreased BMD and increased serum P1NP in comparison with the control group | (20) |
| onion juice | 100 mL of onion juice daily | 11 weeks | 24 postmenopausal women | 40-80 | health | RCT | placebo | anthropometric measurements and blood samples were collected | the BMD of three postmenopausal women was mildly improved. Significant changes in ALP levels, free radicals, TEAC, and various antioxidants. | (21) |
| citrus fruit biscuits | 500 g hesperidin ± 500 mg Ca supplement daily | 3 administrations of 50 days and 3 washout phases of 50 days | 12 postmenopausal women | mean 66.3 | healthy | double-blind, placebo-controlled, randomized order crossover design | placebo | measure the effect of hesperidin with or without Ca supplementation on bone Ca retention in postmenopausal women. | Ca plus hesperidin, but not hesperidin alone, improved bone Ca retention | (22) |
| curcumin capsules | 80 mg curcumin (CUR) and placebo of Nigella sativa oil (NS), 1,000 mg NS and placebo of CUR, 80 mg CUR and 1,000 mg NS, and both placebos (containing microcrystalline cellulose). | 6 months | 120 postmenopausal women | 50-65 | low BMD (T-score ≤ 1) | RCT, triple-blind | placebo (containing microcrystalline cellulose) | test circulating miRNAs in the blood for miRNA-21, miRNA-422a, and miRNA-503 | the expression level of miRNA-21 increased significantly between the four groups and between the NS and placebo groups | (23) |
| red clover pills | 60 mg isoflavone aglycones/ and probiotics + 1200 mg Ca, 550 mg magnesium + 25 mg calcitriol; daily | 1 year | 78 postmenopausal women | 60-85 | osteopenic | RCT, double-blind, parallel design | placebo | measurement of plasma lipids, BMD by DEXA, and bone turnover markers. | improvement of bone status and estrogen metabolism | (24) |
| red clover tablets | 54 mg genistein + Ca and Vitamin D3 supplementation; daily | 2 years | 200 postmenopausal women | case group 60.0 (57.0–66.0) placebo group 62.0 (58.0–67.8) | glucocorticoid-induced osteoporosis | RCT, double-blind | alendronate (70 mg once a week) | measurement of BMD and bone markers (c-terminal telopeptide, OC, bone-ALP, and sclerostin) | BMD at the anteroposterior lumbar spine increased at 12 and 24 months, but no difference was observed between alendronate and genistein groups in BMD. CTX decreased in both groups, while OC, bone-ALP, and sclerostin showed more significant changes in genistein-treated patients. | (25) |
| red clover tablets | 82 mg total isoflavones or 57.2 mg total isoflavones | 12 weeks | 252 symptomatic menopause women | 45-60 | healthy | RCT, double-blind | placebo | measurement of serum OC and urinary cross-linked N-Tx | bone turnover markers showed no significative differences among groups | (26) |
| red clover pills | soy cotyledon, soy germ, kudzu, and red clover | 50 days | 11 postmenopausal women | 59.8 ± 4.7 (mean 52, 65) | healthy | randomized-order, crossover, blinded trial | positive control of oral 1 mg estradiol combined with 2.5 mg medroxyprogesterone or 5 mg/d oral risedronate | ^41^Ca methodology | genistein-like isoflavones demonstrated a significant but modest ability to suppress net bone resorption | (27) |

**Table 3** Clinical trials on the effects of Dairy products on bone loss.

| **Nutraceutical form** | **Dose** | **Treatment length** | **Population: N** | **Population: age (years)** | **Status** | **Type of trial** | **Control group** | **Evaluation performed** | **Results** | **Ref** |
| --- | --- | --- | --- | --- | --- | --- | --- | --- | --- | --- |
| milk solution | 250 ml of partly skimmed milk, milk proteins, Ca salts and stabilizers, vitamins D and K2 with different compositions among the experimental groups; daily | 18 months | 210 postmenopausal women | 39.2 ± 4.6 | healthy | parallel, randomized, double-blind, and single-center | not indicated | BMC, BMD, T-score, and Z-score, biochemical markers | improvement of bone mass in the lumbar spine, less enhanced bone resorption without any differences among the three different compositions | (28) |
| cheese, servings of skimmed milk, soft plain cheese | 2 x 100 g, daily | 6 weeks | 71 | 56.6 ± 3.9 | moderate risk of osteoporosis fracture | prospective control study | control | measurement of serum TRAP 5b and IGF-I | decrease TRAP 5b and increased IGF-I in the treated group | (29) |

**Table 4**. Clinical trials on the effects of Prebiotics and Probiotics

| **Nutraceutical form** | **Dose** | **Treatment length** | **Population: N** | **Population: age (years)** | **Status** | **Type of trial** | **Control group** | **Evaluations performed** | **Results** | **Ref** |
| --- | --- | --- | --- | --- | --- | --- | --- | --- | --- | --- |
| Bifidobacterium lactis powder | Bifidobacterium animalis subsp. lactisProbio-M8 [Probio-M8] mix with maltodextrin, 1.5 × 10^10 CFU/day + Ca + calcitriol daily | 3 months | 40 postmenopausal women | 62.77 ± 6.00 | osteoporotic | RCT | placebo | BMD and gut microbiota analysis | No significant change in BMD. Improvement of bone metabolism by modulating the gut microbiota. Co-administering Probio-M8 improved bone metabolism, reflected by an increased vitamin D3 level and decreased PTH and procalcitonin levels in serum, increased genes encoding some carbohydrate metabolism pathways | (30) |
| multispecies probiotic supplement + Ca (+ Vitamin D) capsules | Lactobacillus casei 1.3 x 10^10 colony-forming units [CFU], Bifidobacterium longum 5 x 10^10 CFU, Lactobacillus acidophilus 1.5 x 10^10 CFU, Lactobacillus rhamnosus 3.5 x 10^9 CFU, Lactobacillus bulgaricus 2.5 x 10^8 CFU, Bifidobacterium breve 1 x 10^10 CFU, and Streptococcus thermophilus 1.5 x 10^8 CFU per 500 mg; daily | 6 months | 50 postmenopausal women | 50-72 | osteopenic | RCT, double-blind | placebo + 500 mg Ca plus 200 IU vitamin D daily. | BMD, BAP, OC, CTX, deoxypyridinoline, PTH, 25-OH vitamin D, and serum pro-inflammatory TNF-α and IL-1β | Significantly decreased BAP and CTX, serum PTH, and TNF-α | (31) |
| Lactobacillus plantarum 3547 as a homogeneous, creamy, yogurt-like textured food enriched with Ca, vitamin D, vitamin K, vitamin C, zinc, magnesium, L-leucine, and Lactobacillus plantarum 3547 | 1X10^10^/150 g daily | 24 weeks | 78 menopausal women | 50-60 | at risk of osteoporosis or untreated osteopenia | parallel, double-blind RCT with two intervention groups | no enrichment | serum P1NP and BMD increased significantly in the EG group, with a significant decrease in CTX compared to the CG group | Arrest of bone loss | (32) |
| Lactobacillus reuteri suspension | 5x10^9^ colony-forming units (CFU) mixed with maltodextrin, yielding a total daily dose of 1x10^10 CFU of L. reuteri 6475; daily twice | 12 months | 70 postmenopausal women | 75-80 | osteopenic | RCT, double-blind | placebo | evaluation of BMD by DEXA | L. reuteri 6475 reduced loss of total vBMD | (33) |
| Lactulose suspension | 100 ml of water containing 5 or 10 g of lactulose daily | 9 days | 12 >5 postmenopausal women | mean 60.5 (range 56-64) | healthy | randomized, double-blind, crossover design, separated by two 19-day wash-out periods | placebo | fractional Ca absorption in urine by ICP-MS | a significant difference in Ca absorption between the highest dose of lactulose and placebo | (34) |
| FOS and a miscellaneous milk | fortified milk (1000 mg Ca for pre-M women and 1200 mg Ca for PM women, 96 mg magnesium, 2.4 mg zinc, 15 μg vitamin D, 4 g FOS-inulin per day); two glasses daily | 12 weeks | premenopausal women, N = 136 postmenopausal women, N = 121 | premenopausal women, mean 41±5; postmenopausal women, mean 59 ±4 | healthy young and older women | RCT | regular milk (500 mg Ca per day) | serum minerals, bone biochemical markers, and BMD | both treatments reduced bone resorption in young and older women; fortified milk more effectively | (35) |
| Transgalactooligosaccharides (TOS) yogurt drink | 200 mL yogurt drink containing TOS (20 g/d); twice daily | two 9-d treatment periods separated by a 19-d washout period | 12 postmenopausal women | 55- 65, mean 62 | NI, presumably healthy | double-blind, randomized crossover study | 200 mL yogurt drink containing the reference substance, sucrose | greater Ca absorption | Transgalactooligosaccharides stimulated Ca absorption | (36) |
| Bacillus subtilis C-3102 tablets | 3.4 × 10^9^/tablet, daily | 24 weeks | 76 postmenopausal women | 50-69 | healthy | RCT, double-blind | placebo | BMD, uNTx, TRACP-5b | Significantly increased BMD; significantly lower uNTX; modulation of the gut microbiota | (37) |

**Table 5**. Clinical trials on the effects of Lipids on bone loss.

| **Dose** | **Treatment length** | **Population** | **Population: age (years)** | **status** | **Type of trial** | **Control group** | **Evaluations performed** | **Results** | **Reference** |
| --- | --- | --- | --- | --- | --- | --- | --- | --- | --- |
| 400 mg DHA +1200 mg Ca carbonate with vitamin D 3 1000 IU daily | 1 year | 36 females, 4 males | mean 59.2 | osteopenic | pilot RCT | placebo + A +1200 mg Ca carbonate with vitamin D 3 1000 IU daily. | serum CTX, questionnaire for tolerability and acceptability | no effects | (38) |
| 900 mg n-3 daily | 6 months | 2 postmenopausal women | treatment 60±5.6 control 63±8.92 | osteoporotic | RCT | placebo | serum levels of OC, bone alkaline phosphatase (BAP), Ca, vitamin D, and parathormone, and urine concentration of Pyd | n-3 fatty acids decreased bone resorption but not significantly affect bone formation | (39) |
| 1) EVOO 50 g or more; daily 2) mixed nuts 30 g; daily | 5.2 years | 870 (female + male) | men 55-80 women 60-80 | cardiovascular risk | observational cohort study nested in the PREDIMED trial | low-fat diet | annual review of patients' medical records | reduced risk of osteoporotic fractures | (40) |

**Table 6**. Clinical trials on the effects of Melatonin on bone loss.

| **Nutraceutical form** | **Dose** | **Treatment length** | **Population** | **Population: age (years)** | **Status** | **Type of trial** | **Control group** | **Evaluations performed** | **Results** | **Reference** |
| --- | --- | --- | --- | --- | --- | --- | --- | --- | --- | --- |
| melatonin tablet | 1 mg (N = 20) and 3 mg (N = 20), nightly | 1 year | 81 postmenopausal women | mean 63 range 56-73 | osteopenic | RCT, double-blind | placebo (N = 41) | DEXA, QCT, HR-pQCT | dose-dependent manner increased BMD at the femoral neck; high-dose melatonin increased vBMD in the spine | (41) |
| melatonin | 3 mg (N = 13), nightly | 6 months | 18 postmenopausal women | 45-54 | osteopenic | RCT, double-blind | placebo (N = 5) | BMD by ultrasound; bone turnover marker (OC and NTX) levels in serum | no significant changes in BMD, NTX, or OC between groups | (42) |

**References**

1. Jehle S, Zanetti A, Muser J, Hulter HN, Krapf R. Partial neutralization of the acidogenic western diet with potassium citrate increases bone mass in postmenopausal women with osteopenia. Journal of the American Society of Nephrology. 2006 Nov;17(11):3213–22.

2. Gregory NS, Kumar R, Stein EM, Alexander E, Christos P, Bockman RS, et al. Potassium citrate decreases bone resorption in postmenopausal women with osteopenia: A randomized, double-blind clinical trial. Endocrine Practice. 2015 Dec 1;21(12):1380–6.

3. Granchi D, Caudarella R, Ripamonti C, Spinnato P, Bazzocchi A, Massa A, et al. Potassium citrate supplementation decreases the biochemical markers of bone loss in a group of osteopenic women: The results of a randomized, double-blind, placebo-controlled pilot study. Nutrients. 2018 Sep 12;10(9).

4. Aydin H, Deyneli O, Yavuz D, Gözü H, Mutlu N, Kaygusuz I, et al. Short-term oral magnesium supplementation suppresses bone turnover in postmenopausal osteoporotic women. Biol Trace Elem Res. 2010 Feb;133(2):136–43.

5. Heaney RP, Recker RR, Watson P, Lappe JM. Phosphate and carbonate salts of calcium support robust bone building in osteoporosis. American Journal of Clinical Nutrition. 2010 Jul 1;92(1):101–5.

6. Radford LT, Bolland MJ, Mason B, Horne A, Gamble GD, Grey A, et al. The Auckland calcium study: 5-year post-trial follow-up. Osteoporosis International. 2014 Jan;25(1):297–304.

7. Elam ML, Johnson SA, Hooshmand S, Feresin RG, Payton ME, Gu J, et al. A calcium-collagen chelate dietary supplement attenuates bone loss in postmenopausal women with osteopenia: A randomized controlled trial. J Med Food. 2015 Mar 1;18(3):324–31.

8. Albertazzi P, Steel SA, Howarth EM, Purdie DW. Comparison of the effects of two different types of calcium supplementation on markers of bone metabolism in a postmenopausal osteopenic population with low calcium intake: A double-blind placebo-controlled trial. Climacteric. 2004 Mar;7(1):33–40.

9. Ebeling PR, Wark JD, Yeung S, Poon C, Salehi N, Nicholson GC, et al. Effects of Calcitriol or Calcium on Bone Mineral Density, Bone Turnover, and Fractures in Men with Primary Osteoporosis: A Two-Year Randomized, Double Blind, Double Placebo Study. J Clin Endocrinol Metab [Internet]. 2001 Sep;86(9):4098–103. Available from: https://academic.oup.com/jcem/article-lookup/doi/10.1210/jcem.86.9.7847

10. Prince RL, Devine A, Satvinder; Dhaliwal S, Dick IM. Effects of Calcium Supplementation on Clinical Fracture and Bone Structure Results of a 5-Year, Double-blind, Placebo-Controlled Trial in Elderly Women.

11. Ciria-Recasens M, Blanch-Rubió J, Coll-Batet M, Del Pilar Lisbona-Pérez M, Díez-Perez A, Carbonell-Abelló J, et al. Comparison of the Effects of Ossein-Hydroxyapatite Complex and Calcium Carbonate on Bone Metabolism in Women with Senile Osteoporosis.

12. Nielsen FH, Lukaski HC, Johnson LK, Roughead ZK. Reported zinc, but not copper, intakes influence whole-body bone density, mineral content and T score responses to zinc and copper supplementation in healthy postmenopausal women. British Journal of Nutrition. 2011 Dec 28;106(12):1872–9.

13. Mahdaviroshan M, Golzarand M, Taramsari MR, Mahdaviroshan M. Effect of zinc supplementation on serum zinc and calcium levels in postmenopausal osteoporotic women in Tabriz, Islamic Republic of Iran. East Mediterr Health J [Internet]. 2013 Mar;19(3):271–5. Available from: http://www.ncbi.nlm.nih.gov/pubmed/23879079

14. Hooshmand S, Brisco JRY, Arjmandi BH. The effect of dried plum on serum levels of receptor activator of NF-κB ligand, osteoprotegerin and sclerostin in osteopenic postmenopausal women: A randomised controlled trial. British Journal of Nutrition. 2014 Jul 14;112(1):55–60.

15. Hooshmand S, Kern M, Metti D, Shamloufard P, Chai SC, Johnson SA, et al. The effect of two doses of dried plum on bone density and bone biomarkers in osteopenic postmenopausal women: a randomized, controlled trial. Osteoporosis International. 2016 Jul 1;27(7):2271–9.

16. Mozaffari-Khosravi H, Hesabgar HAS, Owlia MB, Hadinedoushan H, Barzegar K, Fllahzadeh MH. The effect of garlic tablet on pro-inflammatory cytokines in postmenopausal osteoporotic women: A randomized controlled clinical trial. J Diet Suppl. 2012 Dec;9(4):262–71.

17. Wong RHX, Thaung Zaw JJ, Xian CJ, Howe PRC. Regular Supplementation With Resveratrol Improves Bone Mineral Density in Postmenopausal Women: A Randomized, Placebo-Controlled Trial. Journal of Bone and Mineral Research. 2020 Nov 1;35(11):2121–31.

18. Corbi G, Nobile V, Conti V, Cannavo A, Sorrenti V, Medoro A, et al. Equol and Resveratrol Improve Bone Turnover Biomarkers in Postmenopausal Women: A Clinical Trial. Int J Mol Sci. 2023 Aug 1;24(15).

19. Hodges JK, Maiz M, Cao S, Lachcik PJ, Peacock M, McCabe GP, et al. Moderate consumption of freeze-dried blueberry powder increased net bone calcium retention compared with no treatment in healthy postmenopausal women: a randomized crossover trial. American Journal of Clinical Nutrition. 2023 Aug 1;118(2):382–90.

20. Nosal BM, Sakaki JR, Macdonald Z, Mahoney K, Kim K, Madore M, et al. Blackcurrants Reduce the Risk of Postmenopausal Osteoporosis: A Pilot Double-Blind, Randomized, Placebo-Controlled Clinical Trial. Nutrients. 2022 Dec 1;14(23).

21. Law YY, Chiu HF, Lee HH, Shen YC, Venkatakrishnan K, Wang CK. Consumption of onion juice modulates oxidative stress and attenuates the risk of bone disorders in middle-aged and post-menopausal healthy subjects. Food Funct. 2016 Feb 1;7(2):902–12.

22. Martin BR, McCabe GP, McCabe L, Jackson GS, Horcajada MN, Offord-Cavin E, et al. Effect of hesperidin with and without a calcium (calcilock) supplement on bone health in postmenopausal women. Journal of Clinical Endocrinology and Metabolism. 2016 Mar 1;101(3):923–7.

23. Farshbaf-Khalili A, Farajnia S, Pourzeinali S, Shakouri SK, Salehi-Pourmehr H. The effect of nanomicelle curcumin supplementation and Nigella sativa oil on the expression level of miRNA-21, miRNA-422a, and miRNA-503 gene in postmenopausal women with low bone mass density: A randomized, triple-blind, placebo-controlled clinical trial with factorial design. Phytotherapy Research. 2021 Nov 1;35(11):6216–27.

24. Norman M, Lambert T, Thybo CB, Lykkeboe S, Rasmussen LM, Frette X, et al. Combined bioavailable isoflavones and probiotics improve bone status and estrogen metabolism in postmenopausal osteopenic women: a randomized controlled trial. Am J Clin Nutr [Internet]. 2017;106:909–29. Available from: https://doi.org/10.3945/ajcn.

25. Squadrito F, Imbalzano E, Rottura M, Arcoraci V, Pallio G, Catalano A, et al. Effects of genistein aglycone in glucocorticoid induced osteoporosis: A randomized clinical trial in comparison with alendronate. Biomedicine and Pharmacotherapy. 2023 Jul 1;163.

26. Knudson Schult TM, Ensrud KE, Blackwell T, Ettinger B, Wallace R, Tice JA. Effect of isoflavones on lipids and bone turnover markers in menopausal women. Maturitas. 2004 Jul 15;48(3):209–18.

27. Weaver CM, Martin BR, Jackson GS, McCabe GP, Nolan JR, McCabe LD, et al. Antiresorptive effects of phytoestrogen supplements compared with estradiol or risedronate in postmenopausal women using 41Ca methodology. Journal of Clinical Endocrinology and Metabolism. 2009;94(10):3798–805.

28. Barnuevo MD, Marhuenda J, Aldeguer M, Abellán MS, Zafrilla Rentero P, Contreras CJ, et al. In vivo randomized trial of three marketed milk preparations enriched with calcium and vitamins (D and K) on bone mass and bone turnover markers from biological fluids in premenopausal caucasian women. Nutr Hosp. 2018;35(5):1174–85.

29. Bonjour JP, Benoit V, Rousseau B, Souberbielle JC. Consumption of vitamin D-and calcium-fortified soft white cheese lowers the biochemical marker of bone resorption TRAP 5b in postmenopausal women at moderate risk of osteoporosis fracture. Journal of Nutrition. 2012 Apr 1;142(4):698–703.

30. Zhao F, Guo Z, Kwok LY, Zhao Z, Wang K, Li Y, et al. Bifidobacterium lactis Probio-M8 improves bone metabolism in patients with postmenopausal osteoporosis, possibly by modulating the gut microbiota. Eur J Nutr. 2023 Mar 1;62(2):965–76.

31. Jafarnejad S, Djafarian K, Fazeli MR, Yekaninejad MS, Rostamian A, Keshavarz SA. Effects of a Multispecies Probiotic Supplement on Bone Health in Osteopenic Postmenopausal Women: A Randomized, Double-blind, Controlled Trial. J Am Coll Nutr. 2017 Oct 3;36(7):497–506.

32. Morato-Martínez M, López-Plaza B, Santurino C, Palma-Milla S, Gómez-Candela C. A dairy product to reconstitute enriched with bioactive nutrients stops bone loss in high-risk menopausal women without pharmacological treatment. Nutrients. 2020 Aug 1;12(8):1–15.

33. Nilsson AG, Sundh D, Bäckhed F, Lorentzon M. Lactobacillus reuteri reduces bone loss in older women with low bone mineral density: a randomized, placebo-controlled, double-blind, clinical trial. J Intern Med. 2018 Sep 1;284(3):307–17.

34. Van Den Heuvel EGHM, Muijs T, Van Dokkum W, Schaafsma G. Lactulose stimulates calcium absorption in postmenopausal women. Journal of Bone and Mineral Research. 1999;14(7):1211–6.

35. Kruger MC, Chan YM, Kuhn-Sherlock B, Lau LT, Lau CC, Chin YS, et al. Differential effects of calcium- and vitamin D-fortified milk with FOS-inulin compared to regular milk, on bone biomarkers in Chinese pre- and postmenopausal women. Eur J Nutr. 2016 Aug 1;55(5):1911–21.

36. van den Heuvel EGHM, Muijs T, Schoterman MHC. Transgalactooligosaccharides Stimulate Calcium Absorption in Postmenopausal Women. J Nutr. 2000 Dec;130(12):2938–42.

37. Takimoto T, Hatanaka M, Hoshino T, Takara T, Tanaka K, Shimizu A, et al. Effect of Bacillus subtilis C-3102 on bone mineral density in healthy postmenopausal Japanese women: a randomized, placebo-controlled, double-blind clinical trial [Internet]. Available from: http://greengenes.

38. Vanlint SJ, Ried K. Efficacy and tolerability of calcium, vitamin D and a plant-based omega-3 oil for osteopenia: A pilot RCT. Maturitas. 2012 Jan;71(1):44–8.

39. Sharif PS, Asalforoush M, Ameri F, Larijani B, Abdollahi M. The effect of n-3 fatty acids on bone biomarkers in Iranian postmenopausal osteoporotic women: A randomized clinical trial. Age (Omaha). 2010 Jun;32(2):179–86.

40. García-Gavilán JF, Bulló M, Canudas S, Martínez-González MA, Estruch R, Giardina S, et al. Extra virgin olive oil consumption reduces the risk of osteoporotic fractures in the PREDIMED trial. Clinical Nutrition. 2018 Feb 1;37(1):329–35.

41. Amstrup AK, Sikjaer T, Heickendorff L, Mosekilde L, Rejnmark L. Melatonin improves bone mineral density at the femoral neck in postmenopausal women with osteopenia: a randomized controlled trial. J Pineal Res. 2015 Sep 1;59(2):221–9.

42. Kotlarczyk MP, Lassila HC, O’Neil CK, D’Amico F, Enderby LT, Witt-Enderby PA, et al. Melatonin osteoporosis prevention study (MOPS): A randomized, double-blind, placebo-controlled study examining the effects of melatonin on bone health and quality of life in perimenopausal women. J Pineal Res. 2012 May;52(4):414–26.
